# Cases of Bad Vaccination in the Army

**Published:** 1864-04

**Authors:** Geo. O. Smith

**Affiliations:** Asst.-Surgeon 53d Regiment Ill. Vols.


ARTICLE XVII.

### CASES OF BAD VACCINATION IN THE ARMY.

By GEO. O. SMITH, M.D., Asst.-Surgeon 53d Regt. Ill. Vol.

On the 3d of February, 1864, I was ordered to assume charge of the convalescent camp of the 4th Division, 17th Army Corps, established at Hebron, Miss., eight miles east of Vicksburg.

I found in camp thirty-one cases of vaccination assuming a most unusual appearance. It appears from the history of the cases that on the 15th of December, 1863, two privates (brothers) went into the city of Vicksburg, and found another soldier, whom they represented as a man of bad habits, and who showed marks of dissipation. From him they were vaccinated. A pustule was formed, but instead of maturating, it degenerated into a corroding ulcer, with other symptoms to be described hereafter. From these two men twenty-nine others of the same regiment (23d Indiana) were vaccinated about the 25th of December. Ten of these had been vaccinated before, and had a good mark on their arms. Every one of these twenty-nine took the vaccination; the puncture beginning to inflame immediately after the operation—those who had been vaccinated before taking as readily as the others. These cases all assumed pretty much the same appearance, with the same symptoms, some being more aggravated than others, and a description of any two would be a history of all.

These cases came under my notice on the 4th of February, 1864, at which time Corporals BENJ. DODD and J. MONKS, of Company C, 23d Indiana, came to me and wanted me to look at their arms. On examination of BENJ. DODD, I found his right arm much swollen from the shoulder to the hand, indurated from previous infiltration of lymph. A dark appearance pervaded the whole surface. The skin was abraded about the point of the elbow the size of a man's hand; the tissues beneath were rough, ragged, dry, and scarlet red. At the point of vaccination on the arm was a dark, foul ulcer, an inch and a-half in diameter, and a-half inch deep, with raised, thickened edges. On the fore-arm were two small ulcers a-half inch in diameter, a line in depth, with raised, thickened, indurated edges, having the appearance of venereal chancres. The axillary glands were greatly swollen and indurated, the skin having a dark purple color.

The case of J. MONKS was as follows:—A large foul ulcer like the one described above at the point of vaccination. Two inches below was a well-marked chancre in size and appearance like those described above.

On the fore-arm of DODD, in the axilla, was an abscess, from which I drew one half pint of thin pink-colored pus. Small, red, and hard pimples, the size of a small pea, pervaded the whole face and back.

In most of these cases there has been from the beginning well-marked hectic fever, with inability to sleep at night, and wandering pains in the back and limbs, with loss of appetite. Conjunctivitis accompanied most of the cases, and several had inflammation of the Schniderian membrane. All are and have been unable to do duty.

On the 18th of February, I sent most of these cases to the General Hospital, and so passed from under my care.

